# Addressing mental and physical fatigue in major abdominal surgery by incorporating muscle stretches and hydration mini breaks

**DOI:** 10.1308/rcsann.2024.0120

**Published:** 2025-06-17

**Authors:** J Franklyn, V Sharma, SS Reddy, H Kaur, D Wildash, J Bell, SP Dayal, A Tzivanakis, F Mohamed, BJ Moran, T Cecil

**Affiliations:** Basingstoke and North Hampshire Hospital, UK

**Keywords:** Human factors, Minibreaks, Major surgery

## Abstract

**Introduction:**

The purpose of this quality improvement project (QIP) was to identify factors contributing to mental and physical fatigue in major abdominal surgery and to attempt to mitigate the same by incorporating mini hydration breaks with targeted muscle stretches at regular intervals.

**Methods:**

This prospective QIP was conducted in the Peritoneal Malignancy Unit of a national referral centre for peritoneal malignancy-related diseases between February and April 2022. Only procedures lasting longer than four hours were included and all theatre personnel were invited to participate. A baseline survey was conducted to ascertain the impact of mental and physical fatigue. Subsequently, a cross-over study design was utilised; for the first four weeks the procedure was performed with no breaks. This was followed with four weeks of intervention (hydration breaks and muscle stretches). Validated questionnaires (pain scores, occupational fatigue inventory and surgical task load measurement) were used to measure perceived physical and mental fatigue.

**Results:**

Over half (58%) of the 34 participants felt that surgical discomfort affected their stamina, posture and ability to concentrate. Work–life balance was affected in 44%, and 17% felt that it affected their sleep pattern with a minority considering shortening their careers. A reduction in the mean pain score at the end of the day in the group who had breaks (2.61 vs 2.16) was noted. There was global improvement in situational stress, distractibility, temporal and mental demands in the group who had regular breaks, amounting to improvements in self-perceived fatigue levels.

**Conclusions:**

Theatre personnel involved in major surgery experience mental and physical stress that adversely affects work–life balance. Regular, short hydration breaks with muscle stretches can help improve mental and physical wellbeing of theatre personnel involved in major abdominal surgery.

## Introduction

Theatre personnel working long hours without regular breaks are likely to be tired, inattentive, dehydrated and ultimately less vigilant.^1^ In addition, performing long operations without mitigation measures can lead to musculoskeletal injury, adversely affect work–life balance and potentially shorten careers.^2^

Recognition of the importance of workplace fatigue, regular hydration and rest breaks has been enshrined in the work culture of workers in the airline industry and professional lorry drivers with reported beneficial results.^3–5^ There is a growing body of evidence encouraging the adoption of rest breaks in the operating theatre; however, for a plethora of reasons ranging from surgical culture to lack of awareness, rest breaks have not been established in standard operating procedure protocols. A recent multi-centred cohort study has also suggested that performing targeted muscle stretches at periodic intervals intraoperatively can decrease musculoskeletal pain.^6^

Whereas most studies have looked at mandatory operative breaks from either the surgical or anaesthetic perspective, this paper explores the impact of incorporating hydration breaks on a global level for the entire theatre team performing complex abdominal surgery.

The purpose of this quality improvement project (QIP) was to identify the factors contributing to mental and physical fatigue in major abdominal surgery and to attempt to mitigate mental and physical fatigue by incorporating mini hydration breaks with targeted muscle stretches at regular intervals during major surgery.

## Methods

### Design and setting

This prospective, single-centre QIP was conducted in the Peritoneal Malignancy Unit of a national referral centre for peritoneal malignancy-related diseases between February and April 2022 after obtaining permission from the research and development department. All patients underwent cytoreductive surgery and hyperthermic intra-peritoneal chemotherapy (CRS and HIPEC). Since most procedures last a median of eight hours, two consultant surgeons are assigned for each procedure with a recommended lunch break for all theatre personnel.

Eligible participants included all theatre personnel involved in peritoneal malignancy operative procedures. Unlike earlier studies where the duration of surgery was heterogeneous, in this study only procedures likely to last longer than four hours were included.

A cross-over study design was utilised, wherein, for the first four weeks, the operative procedure was performed with no breaks. This was followed with four weeks of intervention (hydration breaks and muscle stretches). Both control and intervention weeks had pre- and post-procedure validated questionnaires to objectively measure physical and mental fatigue as well as surgical task load.

### Initial questionnaire and baseline phase

**Figure 1:**
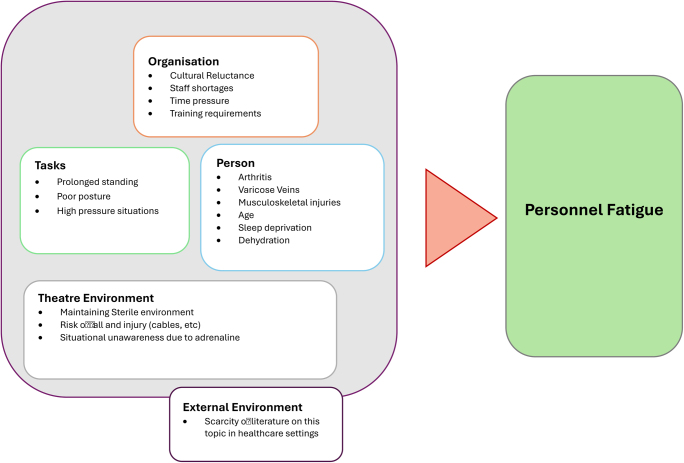
SEIPS conceptual model to perform a root cause analysis of theatre personnel fatigue. SIEPS = System engineered initiative for patient safety

**Figure 2:**
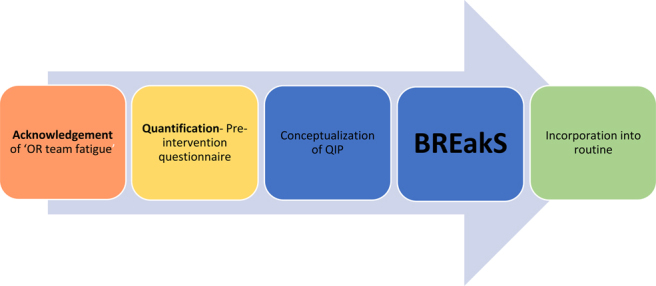
Flowchart of study from inception to incorporation into theatre routine BREakS = Basingstoke Rest and Exercise Study; OR = operating room; QIP = quality improvement project

Theatre personnel completed an initial questionnaire (Appendix 1) via an online form to understand the extent of their physical and mental fatigue, along with suggestions to mitigate fatigue. The results of this questionnaire were analysed and a literature review carried out to find the most suitable intervention (Table 1 and 2). Based on the results of the initial questionnaire and the literature review, a root cause analysis was performed using the system engineered initiative for patient safety (SEIPS) conceptual model and a decision was made to proceed with the QIP (Figures 1 and 2).^7^

**Table 1:** Baseline assessment of mental and physical wellbeing

	Number (%)
Job title	
Consultant surgeon	8(23.5)
Anaesthetic Consultant	3(8.8)
Registrar/Fellow	11(32.5)
Theatre nurse	8(23.5)
SHO	3(8.8)
ODP	1(2.9)
Age	
<30 years	2(5.9)
30–40 years	14(41.2)
40–50 years	13(38.2)
50–60 years	3(8.8)
60+ years	2(5.9)
Theatre experience in years	
<5	6(17.6)
5–10	5(14.7)
10–20	15(44.1)
20+	8(23.5)
Pre-existing MSK problem	
Yes	8(23.5)
No	24(70.6)
Prefer not to say	2(5.9)
Has surgical discomfort affected the following	
Balance	6(17.6)
Concentration	12(35.4)
Mobility	8(23.5)
Object localisation	2(5.9)
Posture	20(58.8)
Stamina	11(32.4)
Pain	4(11.8)
How do you think operative fatigue affects you?	
Adversely affects work/life balance	15(44.1)
Alters sleeping pattern	6(17.6)
I have considered shortening/changing career	4(11.8)
Pain	3(8.8)
How often do you have muscle/joint pain at the end of the procedure?	
Every time I operate	3(8.8)
Once a month	17(50)
Once a week	8(23.5)
Rarely	4(11.8)
Never	2(5.9)
Do you feel dehydrated and drained at the end of an operating session?	
Yes	16(47.1)
No	4(11.8)
Maybe/depends on the case	14(41.2)

MSK = musculoskeletal; ODP = operating department practitioner; SHO = senior house officer

**Table 2:** Preliminary survey of strategies employed to address fatigue by theatre staff

How do you deal with operative discomfort?	Taking a break	10(29.4)
	Ignore it	16(47.1)
	Change operative/patient position	9(26.5)
	Build general fitness by exercising outside of work	21(61.8)
	Other	4(11.8)
Do you routinely implement risk reducing strategies in the OT to alleviate musculoskeletal issues? e.g. stretching, taking breaks, etc	Yes	8(23.5)
	No	19(55.9)
	Occasionally	7(20.6)
Do you routinely stretch or exercise muscle groups outside the context of work in a gym or at home?	Yes	18(52.9)
	No	9(26.5)
	Rarely	7(20.6)

OT = operating theatre

### Control month 1—no breaks or exercises

During the first month, all procedures were carried out without any mandatory breaks for water or exercises. Pre- and post-procedure pain scores, the surgical task load index (SURG-TLX) and the Swedish occupational fatigue inventory (SOFI) were used to objectify the results (Table 3).

**Table 3:** Difference in pain scores measured before and during the intervention

Body parts	Difference in Mean pain score before breaks (Post op–Pre op=difference)	Mean pain score after breaks (Post op–Pre op=difference)	Improvement
Neck	2.66–1.48=1.18	1.98–1.67=0.31	0.87
Shoulder	2.75–1.67=1.08	2.09–1.66=0.43	0.65
Upper back	2.54–1.37=1.17	2.42–1.84=0.58	0.59
Lower back	3.24–2.00=1.24	2.53–2.01=0.52	0.72
Arms	2.06–1.20=0.86	1.65–1.37=0.28	0.58
Wrists	2.15–1.29=0.86	1.94–1.55=0.39	0.47
Knees	3.38–1.90=1.48	2.27–1.79=0.48	1.00
Ankle	3.13–1.70=1.43	2.37–1.62=0.75	0.68
Mean pain score	2.61–1.58=1.03	2.16–1.68=0.46	0.57

Post op = post operative; Pre op = pre operative

### Intervention month 2—intervention month with breaks for water and exercise twice during the operation

The exercise breaks were designed with the local physiotherapy team and were based on the exercises discussed by Park *et al*.^15^ The routine was designed to be adequately short, with just over a minute required for the entire sequence and in a manner to ensure sterility of scrubbed theatre personnel, i.e. hands visible always, enough space between people. The routine was simplified and made memorable and not distracting by ensuring the routine went from head down to toes. To avoid slippage on any potentially wet theatre surface, the exercise routine ensured both feet on ground for greater stability of the participants.

A demonstrative video was created and circulated to all theatre personnel before the intervention weeks to allow them to familiarise themselves with the exercises (Supplemental video 1). Numerous posters were also placed on the wall of the theatre to allow staff to follow the routine even without the physiotherapy team leading the break.

**Figure 3:**
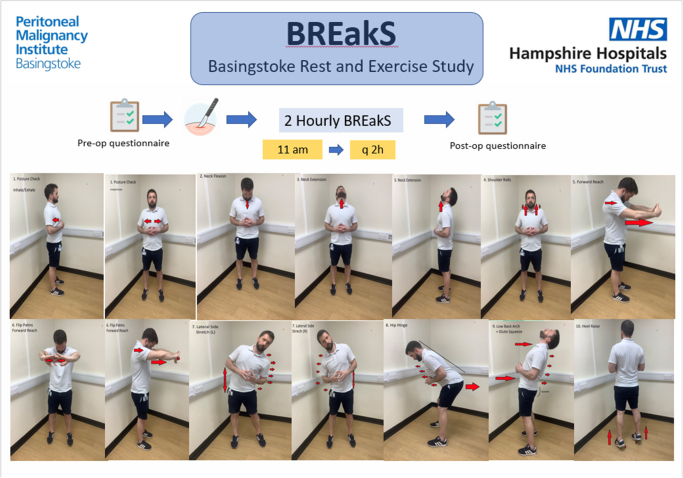
Schematic poster showing BREakS exercise sequence BREakS = Basingstoke Rest and Exercise Study

After the exercise break, a hydration break was also taken and all theatre personnel were encouraged to drink water from a sealed cup using a straw. The total duration of these short breaks was around two minutes. Breaks were held approximately twice during a surgical procedure, with the first break taken around two hours into the operation. The breaks were coordinated by the consultant and fellow in the theatre and repeated in approximately two hours when patient safety permitted (Figure 3).

### Assessment tools

All theatre personnel participating in these procedures completed pain scores pre- and post-operatively (Appendix 1). The mean difference in pain scores (pre-operative MSK pain and post-procedure MSK pain) was compiled. The mean difference in pain score between the two arms of the study, i.e. control and intervention arm, was analysed.

In addition, all participants completed the following questionnaires post-operatively:

• SURG-TLX, which assesses the impact of stress on the perceived demands of surgeons and other theatre personnel in completing a surgical task^8^;
• SOFI, a tool that assesses work-related fatigue under five domains (sleepiness, lack of motivation, physical discomfort, physical exertion and lack of energy), with each of these domains having five questions.^9^

These two questionnaires were filled in only at the end of the operating day and the changes in scores were compared between the two arms (control and intervention).

The forms were made available in print form in the operating room and all theatre staff were encouraged to fill the pre-operative section before the procedure began and the post-operative section at the end. This was reinforced by including a reminder in the World Health Organisation check-in and check-out. All forms were submitted at the end of the day into a confidential box (Appendix 2).

### Data analysis

All results were analysed using SPSS (IBM Corp. Released 2016. IBM SPSS Statistics for Windows, Version 24.0. Armonk, NY: IBM Corp.). The average change in pain from pre- to post-operation was calculated for all body parts. As the raw data were normally distributed, an independent *t*-test was used to calculate whether the difference from control weeks to intervention weeks was statistically significant. Independent *t*-tests were also used for calculating significance in change in average SOFI scores for each measure as well as for surgical task load measures. For all analysis a *p*-value less than 0.05 was considered statistically significant.

## Results

### Baseline survey of theatre personnel

A total of 34 theatre personnel completed the questionnaire that assessed the baseline mental and physical wellbeing. Most participants were in their 40s with at least ten years of operating theatre experience. The physical and mental effect of performing complex surgery along with its effect on work–life balance is outlined in Table 1. The strategies employed by theatre personnel to deal with surgical fatigue are depicted in Table 2.

### Impact of hydration breaks and muscle stretches

A total of 41 operations were performed, 20 procedures without breaks and 21 with BREakS (Basingstoke Rest and Exercise Study). The procedures lasted a median of seven hours (range 4.5–11h). The median operative time was similar between the two groups. The results of this project suggest that there were global improvements in musculoskeletal discomfort after structured breaks were introduced. Most practitioners perceived an improvement in their mental focus and felt less distractible as reflected by the improvement in surgical task load index (Table 4).

**Table 4:** Surgical task load*

Surgical task load measure (n=88)	Before breaks mean	After breaks mean	Difference
Mental demand	4.51	3.90	0.61
Physical demand	4.76	3.90	0.86
Temporal demand	3.70	2.94	0.76
Task complexity	5.41	4.37	1.04
Situational stress	3.48	2.50	0.98
Distracted	3.5	2.69	0.81

*Operating theatre practitioners’ perception of their ability to complete a task at the end of the operating day.

The SOFI reports a multi-dimensional assessment of procedure-related fatigue. Table 5 represents an improvement in the overall fatigue levels of the theatre personnel.

**Table 5:** SOFI measure before and after breaks were incorporated into standard care

	Parameters	Pre-intervention	Post-intervention	Difference
Physical exertion	Palpitations	0.40	0.17	0.23
	Lack of concern	0.76	0.44	0.32
	Sweaty	0.62	1.16	−0.54
Physical discomfort	Tense muscles	1.66	1.57	0.11
	Numbness	0.91	0.45	0.46
	Stiff joints	1.44	1.20	0.24
	Aching	2.00	1.64	0.36
Sleepiness	Falling asleep	0.61	0.42	0.19
	Drowsy	0.83	0.42	0.41
	Yawning	1.14	0.73	0.41
Lack of motivation	Lack of concern	0.75	0.37	0.38
	Passive	0.89	0.34	0.55
	Uninterested	0.65	0.34	0.31
Lack of energy	Worn out	1.61	1.23	0.38
	Over-worked	1.41	0.95	0.46

SOFI = Swedish occupational fatigue index

## Discussion

The results of this study bring to attention the levels of stress theatre personnel endure in pursuit of optimal patient care. The baseline questionnaire that was sent out to all theatre personnel revealed that 47% felt dehydrated at the end of the procedure, with musculoskeletal discomfort affecting their posture, stamina and ability to concentrate. Most respondents felt that operative fatigue and musculoskeletal pain affected their work–life balance and sleep pattern, with 11% considering shortening their careers due to these factors. These findings are amplified by the fact that most (55%) theatre personnel do not implement any risk-reducing strategies and 48% of respondents did not stretch or regularly exercise outside of work. Most theatre personnel do not take a break when they have discomfort, and rarely stretch or exercise consciously, due partly due to surgical culture and partly to lack of awareness.^10^

Workplace fatigue has contributed to multiple tragedies ranging from the Chernobyl nuclear disaster to the Spuyten-Duyvil rail derailment.^9,11,12^ Although these may be extreme examples, an exhausted workforce can undoubtedly lead to medical errors.^13^ The findings of the preliminary survey have been echoed by various authors previously.^14,15^ However, most studies have focused on either surgeons or anaesthetists. The results of this study are unique as the focus is on the wellbeing of all theatre personnel, in particular the operating department practitioners and nurses, who are equally affected by fatigue yet underrepresented in studies.^16–18^

With this as the backdrop, a QIP was initiated; after a thorough literature review, we decided to address surgical mental and physical fatigue by incorporating hydration breaks and muscle stretches at pre-determined times during long surgical procedures.

Structured breaks have been shown to be effective in enabling tired and stressed theatre staff to refocus and mentally regroup. This is more important for team members who are assisting or performing passive roles, as research has shown that health professionals who are tired can have ‘micro-sleeps’ lasting a few seconds. A small randomised controlled study reported that introducing ‘micro-pauses’ can improve wellbeing without affecting overall timing of completing a procedure or disrupting flow.^19^ Additionally, there have been renewed efforts to reduce occupational musculoskeletal pain by encouraging desk job workers to perform short stretches, a concept that was incorporated into the operating room by Park *et al.*^16,20^

There was universal ‘buy in’ for the proposal, with all theatre personnel eager to participate in the project, thus reflecting the appetite for such change in a modern operating theatre. Our results revealed global improvement in pain scores measured after the surgery in the month when breaks were implemented. There was also subjective improvement in the ability to complete a surgical task with less fatigue perceived by the theatre personnel, most of whom felt more awake and energetic at the end of a procedure. Given the subjective nature of the tools used to measure fatigue and the single-centre study design, we chose to avoid overanalysis of the results, but rather present this as a proof-of-concept project.

The strengths of this project are as follows: the exercise routine has been simplified and takes into consideration the need for sterility and space in theatre, thus making it reproducible and feasible. Additionally, to our knowledge, this is probably the first time that premeditated sterile ‘hydration breaks’ have been introduced to prevent dehydration of theatre personnel involved in major surgery. In addition, these measures encourage theatre personnel to be open about work-related fatigue and serve to de-stigmatise this topic. This is very important in the context of major surgery, where surgical commitment and human factors have historically been at loggerheads. Another observation we made was that these breaks acted as a conversation starter, which improved communication and decreased operative room tension.

Despite these positive attributes there are certain limitations. First, we performed a ‘before and after’ analysis; the findings of this paper can be explained quite easily as a regression to the mean. Due to the subjective nature of assessment and the ‘buzz’ generated around the project in coffee rooms, the positive findings in the project could be explained by the Hawthorne effect.

Despite these limitations and the obvious need for the results of this study to be validated in multi-centred studies, the concept can be generalised to most minimally invasive and open major surgery. We have not relied on statistical significance to confer practical relevance for obvious reasons. We have also not discussed the effect that the breaks had on operative time or on ‘disruption of flow’ primarily because the breaks were triggered by the consultant when deemed safe, and the median operation time for peritoneal malignancy treatment is around seven hours, and therefore two-minute breaks were unlikely to be hugely consequential.

## Conclusions

Theatre personnel involved in major surgery experience mental and physical stress that adversely affects work–life balance. Short hydration breaks with easy-to-follow muscle stretches can be incorporated into the standard practice of major abdominal surgery, serving to alleviate muscle stiffness and mental fatigue, thereby allowing theatre personnel to focus, regroup and concentrate on the job at hand.
